# Supplementary material for: Complexome profiling of the Chlamydomonas psb28 mutant reveals TEF5 as an early PSII assembly factor
Source: Plant Cell. 2025 Mar 17;37(6):koaf055. doi: 10.1093/plcell/koaf055 (PMC12164590; doi:10.1093/plcell/koaf055)
Supplement: koaf055_Supplementary_Data [file koaf055_supplementary_data.zip › psb28_interactive.zip!/plots/intensity/qualityPlots/peptides/_Cre11.g477500.t1.2.html]

### Cre11.g477500.t1.2

Trivial name:   
SeqCoverage [median]: 10.50  
Mass: 30.25 kDa  
  
 

| Sequence | MissedCleavage | Mass [Da] | wtIntensity | muIntensity | Score |
| --- | --- | --- | --- | --- | --- |
| GDMLKPSVVNTPLVR | 1 | 1624.90 | 0.00 | 1618.77 | 47.84 |
| YDIENLPEK | 0 | 1119.54 | 338.05 | 769.27 | 113.74 |
| YDIENLPEKTDADKDK | 2 | 1892.90 | 0.00 | 845.19 | 46.96 |
|  |  |  | 338.0 | 3233.2 |  |
